# Delayed Stevens–Johnson syndrome induced by combined administration of carbamazepine and botulinum toxin: A case report

**DOI:** 10.1097/MD.0000000000041289

**Published:** 2025-01-17

**Authors:** Weiqian Liu, Bo Gao, Ye Yuan, Shaowei Xie, Shengxian Jiao, Wenhui Feng, Dongrui Yan, Yu Yin

**Affiliations:** a Department of Rehabilitation Medicine, Hebei General Hospital, Shijiazhuang, Hebei, China; b Hebei Provincial Key Laboratory of Cerebral Networks and Cognitive Disorders, Hebei General Hospital, Shijiazhuang, Hebei, China; c Department of Urinary Surgery, Hebei General Hospital, Shijiazhuang, Hebei, China.

**Keywords:** botulinum toxin, carbamazepine, case report, Steven–Johnson syndrome (SJS)

## Abstract

**Rationale:**

Steven–Johnson syndrome (SJS) is characterized by severe illness, rapid progression, and high mortality rates, with the vast majority of cases induced by medications. Botulinum toxin, a neurotoxin produced by Clostridium botulinum, has not been reported in the literature as a causative agent of SJS.

**Patient concerns:**

A 56-year-old male patient, who underwent surgery for cerebral hemorrhage, developed widespread patchy annular papules following the injection of botulinum toxin into the masseter muscle. Some lesions exhibited a target-like appearance, and all major organ systems were affected.

**Diagnoses:**

Consider the delayed SJS induced by the combination of carbamazepine and botulinum toxin.

**Interventions:**

Intravenous administration of methylprednisolone in conjunction with immunoglobulin is indicated. For ocular lesions, topical treatment includes tobramycin-dexamethasone and sodium hyaluronate eye drops; for ulcerated areas, local application of lactulose-iodoquinol is recommended, while non-ulcerated regions should be treated with halometasone ointment topically.

**Outcomes:**

The patient has been discharged, and there has been a noticeable improvement in their symptoms.

**Lessons:**

In order to prevent severe adverse reactions, patients using carbamazepine in conjunction with other medications should be vigilant for the early symptoms of serious drug rashes.

## 1. Introduction

Stevens–Johnson syndrome (SJS) is a severe skin and mucosal reaction, predominantly triggered by medications. It is characterized by the formation of blisters and widespread epidermal detachment, often accompanied by multi-system involvement.^[1]^ Common medications that are frequently associated with severe drug-induced skin reactions include sulfonamides, aminopenicillins, cephalosporins, fluoroquinolones, carbamazepine, phenytoin, phenobarbital, valproic acid, oxicam-class non-steroidal anti-inflammatory drugs, and allopurinol.^[2]^

Botulinum toxin, produced by the bacterium Clostridium botulinum, is a neurotoxin that specifically targets nerve cells. It inhibits the release of acetylcholine at the neuromuscular junction, thereby obstructing the transmission of nerve impulses. This ultimately leads to muscle paralysis and dysfunction.^[3]^

This article reports a case of SJS associated with the combination of carbamazepine and botulinum toxin.

## 2. Case presentation

The patient is a 56-year-old male who was admitted due to impaired mobility in the limbs. The patient was diagnosed on December 8, 2023, with “ruptured posterior communicating artery aneurysm and subarachnoid hemorrhage.” Surgical intervention included “craniotomy for hematoma evacuation, decompressive craniectomy, and clipping of the intracranial aneurysm.” Due to hydrocephalus, on March 21, 2024, the patient underwent laparoscopic ventriculoperitoneal shunt placement and left cranial titanium plate implantation. Postoperatively, the patient has been adhering to a regular regimen of carbamazepine at a dosage of 0.1 g twice daily for epilepsy management and is committed to ongoing rehabilitation therapy.

**Figure 1.**
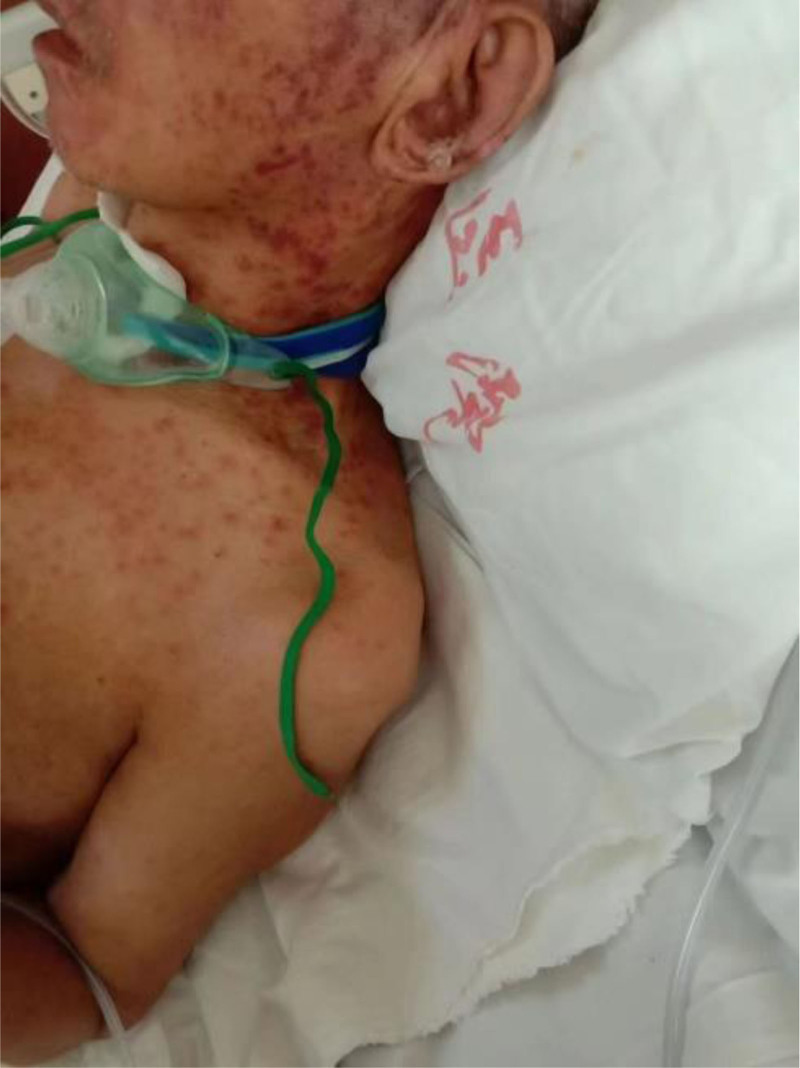
April 19, 2024 the patient presented with scattered red rashes on the face and neck.

**Figure 2.**
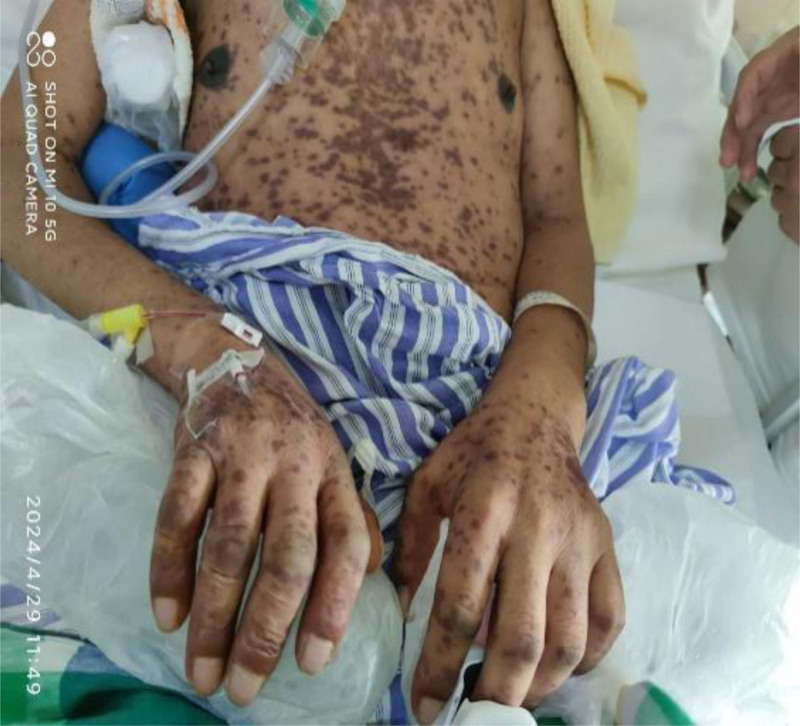
April 29, 2024 the patient presented with severe rash accompanied by ulceration.

**Figure 3.**
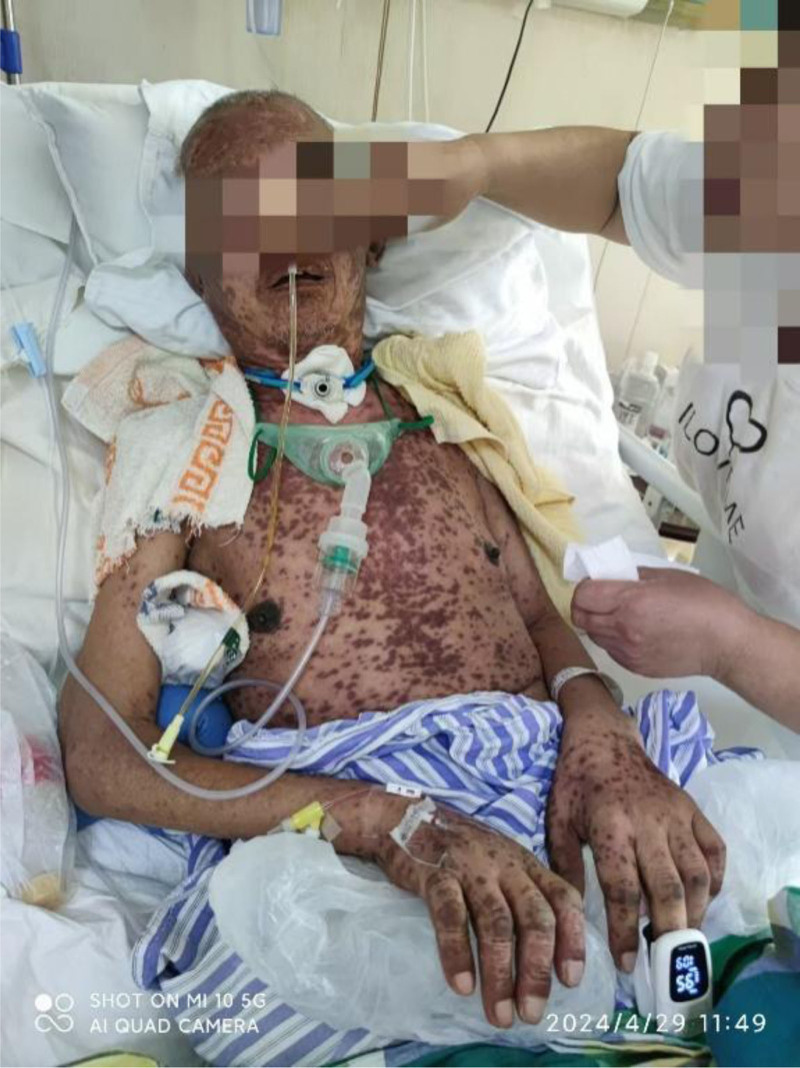
April 29, 2024 the patient presented with severe rash accompanied by ulceration.

**Figure 4.**
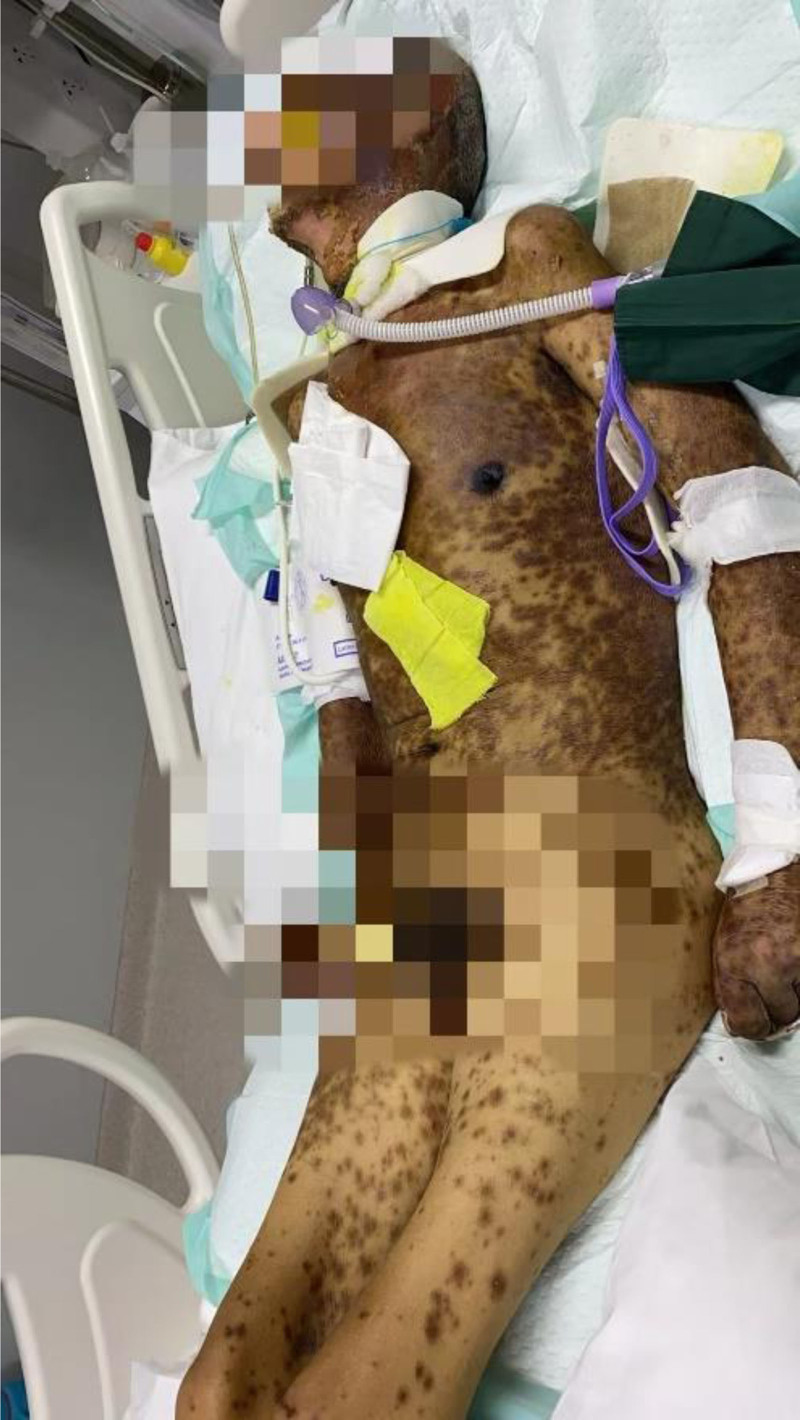
May 5, 2024 the patient’s systemic rash showed significant improvement; however, patchy erythema remained visible on newly formed skin.

**Figure 5.**
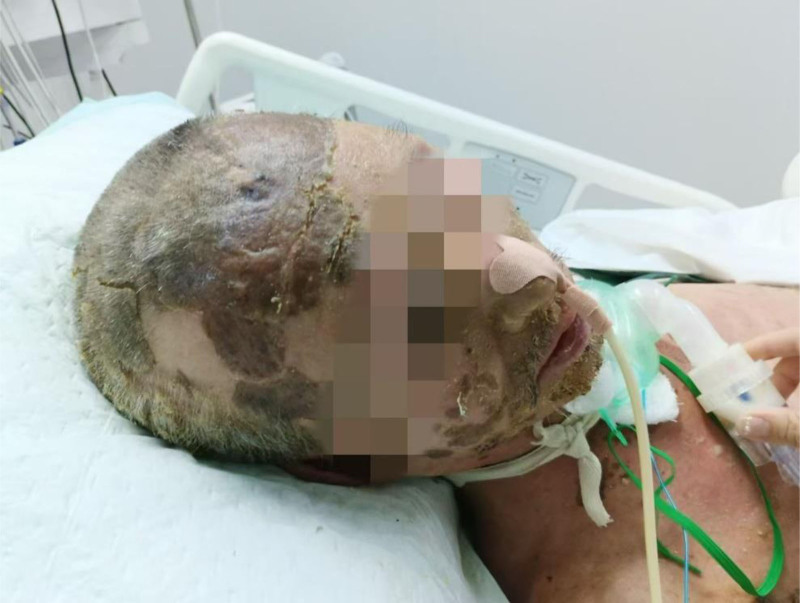
May 14, 2024 the patient’s systemic rash showed significant improvement; however, patchy erythema remained visible on newly formed skin.

On April 19, 2024, the patient presented with scattered red rashes on the face and neck (Fig. 1). A consultation with the dermatology department was requested to consider seborrheic dermatitis. The patient was prescribed topical application of dexamethasone cream. Due to the patient’s severe bruxism, and in order to alleviate the symptoms, a total of 100 units of bilateral masseter botulinum toxin (Botox, Allergan Ireland) were administered under ultrasound guidance on April 22, 2024. After the patient developed an increased number of rashes, multiple patchy annular papules were observed throughout the body, with some lesions exhibiting target-like morphology. A consultation with dermatology was requested to consider a diagnosis of erythema multiforme. Oral symptomatic treatment was initiated with Ebastine and Olopatadine; however, the therapeutic effect was suboptimal. On April 26, 2024, the patient presented with severe rash accompanied by ulceration (Figs. 2 and 3). A intravenous infusion of methylprednisolone at a dosage of 40 mg was administered. A consultation with the dermatology department was requested again, leading to a diagnosis of erythema multiforme. The management plan included: simplification of medication regimen; continuation of methylprednisolone treatment at a dosage of 40 mg, with careful monitoring for potential side effects associated with corticosteroid therapy; application of compound Huangbai solution as wet dressings on ulcerated areas to prevent infection. Please consult the Pharmacy Department. Recommendation: the possibility of adverse drug reactions caused by carbamazepine cannot be excluded. It is advised to discontinue carbamazepine on April 28, 2024. On April 29, 2024, the patient exhibited ocular symptoms characterized by increased secretion and difficulty in opening the eyes. The dosage of methylprednisolone was escalated to 120 mg for intravenous infusion. The consultation opinion from the hospital on April 30, 2024, considers the diagnosis of SJS to be definitive. The patient exhibits involvement of multiple organ systems. This episode is thought to be a result of an individual’s hypersensitive constitution, compounded by carbamazepine and botulinum toxin-induced delayed severe drug eruption. To prevent stress-related ulcers and gastrointestinal bleeding, intravenous administration of sodium omeprazole is recommended. For the ocular lesions observed in the patient, topical treatment with tobramycin-dexamethasone and sodium hyaluronate eye drops is advised. For ulcerated areas, local application of lactate esomeprazole is indicated, while non-ulcerated regions should receive halometasone ointment topically. From April 29, 2024, to May 3, 2024, the patient received a total of 20 g of immunoglobulin for shock therapy. On May 5, 2024, the dosage of methylprednisolone was reduced to 80 mg via intravenous infusion. Subsequently, on May 9, it was further decreased to 40 mg. Following this treatment regimen, the patient’s systemic rash showed significant improvement; however, patchy erythema remained visible on newly formed skin (Figs. 4 and 5). On May 14, the dosage of sodium methylprednisolone succinate was increased to 60 mg for intravenous administration. The patient subsequently improved and was discharged from the hospital.

## 3. Discussion

In recent years, botulinum toxin has gradually been applied in the clinical treatment of post-stroke muscle spasticity as a neurochemical blocking agent. This medication can reduce muscle tone by inhibiting the release of acetylcholine, thereby allowing muscles to return to a relaxed state and alleviating the degree of muscular contraction and tension. Previous literature has not reported cases of SJS associated with botulinum toxin; moreover, the patient had been regularly taking carbamazepine for over one month prior to admission. Notably, scattered red rashes were already present on the patient’s face before the injection of botulinum toxin, indicating that adverse reactions solely attributable to carbamazepine cannot be completely ruled out. However, it is important to note that severe drug eruptions occurred after the administration of botulinum toxin, demonstrating a strong temporal association. According to the Naranjo Adverse Drug Reaction Probability Scale (Table 1), which assesses the likelihood that an adverse reaction is related to a specific medication, this case scored 1, suggesting a possible correlation. We assessed the severity of the patient’s condition at the time of discharge. According to the SCORTEN score, the patient received a score of 2 (Table 2), which predicts a mortality risk of 12% (the corresponding predicted mortality risks for scores ranging from 0 to 7 are as follows: 1%, 4%, 12%, 32%, 62%, 85%, and finally, 95% and 99%).^[4]^ The pharmacological studies of carbamazepine indicate^[5]^ that there is significant individual variability in its pharmacokinetics. Absorption in the human body occurs slowly and irregularly, accompanied by a process of self-induction. The maximum enzyme activity is observed 2 to 4 weeks after administration. In adults, the elimination half-life is prolonged and varies widely, ranging from 25 to 65 hours.^[6]^ Therefore, it is considered that carbamazepine may contribute to the development of delayed severe drug rash induced by botulinum toxin. The interaction between carbamazepine and botulinum toxin may lead to pharmacokinetic interactions, potentially resulting in SJS. Sisodiya et al^[7]^ reported four cases where the addition of levetiracetam exacerbated the toxicity of carbamazepine. However, in these four cases, serum levels of both carbamazepine and its epoxide remained unchanged, prompting the authors to hypothesize that a pharmacodynamic interaction exists between carbamazepine and levetiracetam due to an additive effect leading to increased toxicity symptoms associated with carbamazepine. A similar pharmacodynamic interaction has been observed with lamotrigine; following the addition of lamotrigine, serum levels of both carbamazepine and its epoxide also did not change.^[8]^

**Table 1:** Naranjo ADR evaluation scale.

Related questions	Yes	No	Unknown
1. Is there any conclusive report before this ADR?	1	0	0
2. Do the ADR occur after the use of suspect drugs?	2√	−1	0
3. Do the ADR relieve after drug withdrawal or use of antagonist?	1	0	0
4. Does the ADR recur after reuse of the suspect drug?	2	−1	0
5. Are there other reasons that can cause the ADR independently?	−1√	2	0
6. Does the ADR repeat after the application of placebo?	−1	1	0
7. Does the drug reach toxic concentration in blood or other body fluids?	1	0	0
8. Is the ADR aggravated (relieved) with the increase (decrease) of dose?	1	0	0
9. Has the patient ever been exposed to the same or similar drugs and had similar reactions	1	0	0
10. Is there any objective evidence to confirm the reaction?	1	0	0
Total score			

The total score ≥9 shows that the causal relationship of adverse drug reactions is definite; the total score 5 to 8 is probably or likely to be relevant; the total score 1 to 4 is possible to be relevant; the total score ≤0 is doubtful to be relevant.

ADR = adverse drug reactions.

**Table 2:** Severity-of-illness score for toxic epidermal necrolysis (SCORTEN).

Risk factor	Score	
	0	1
Age√	<40 yr	≥40 yr
Associated cancer	No	Yes
Heart rate (beats/min)	<120	≥120
Serum blood urea nitrogen	≤28 mg/dL (10 mmol/L)	>28 mg/dL (10 mmol/L)
Detached or compromised body surface√	<10%	≥10%
Serum bicarbonate	≥20 mEq/L (≥20 mmol/L)	<20 mEq/L (<20 mmol/L)
Serum glucose	≤250 mg/dL (≤13.88 mmol/L)	>250 mg/dL (>13.88 mmol/L)

The corresponding predicted mortality risks for scores ranging from 0 to 7 are as follows: 1%, 4%, 12%, 32%, 62%, 85%, and finally, 95% and 99%.

SCORTEN = severity-of-illness score for toxic epidermal necrolysis.

SJS is characterized by severe illness, rapid progression, and high mortality rates. It is strongly recommended that clinicians carefully inquire about the patient’s medical history and allergy history before administering antiepileptic drugs such as carbamazepine. Additionally, after the use of other medications, vigilance for prodromal symptoms of severe drug rash should be maintained. Close monitoring of changes in the patient’s condition is essential, with particular attention to signs such as fever, conjunctival injection, gastrointestinal bleeding, and abnormalities in liver and kidney function. Especially when prodromal symptoms like fever, ocular congestion, or pharyngeal discomfort arise, it is crucial to promptly analyze their causes while maintaining a heightened awareness of potential adverse drug reactions. If a drug rash is suspected at any point, the suspected medication should be discontinued immediately; symptomatic and supportive treatment must then be initiated to prevent serious consequences.

## Author contributions

**Conceptualization:** Weiqian Liu, Yu Yin.

**Data curation:** Weiqian Liu.

**Investigation:** Weiqian Liu, Yu Yin.

**Methodology:** Weiqian Liu, Yu Yin.

**Resources:** Weiqian Liu.

**Writing – original draft:** Weiqian Liu.

**Writing – review & editing:** Bo Gao, Ye Yuan, Shaowei Xie, Shengxian Jiao, Wenhui Feng, Dongrui Yan.
